# Synthetic Multiepitope Protein-Based Plasmonic Immunosensor for Rapid, Label-Free Detection of *Cryptococcus* Antibodies in Human Serum

**DOI:** 10.1021/acsinfecdis.5c00315

**Published:** 2025-08-02

**Authors:** Camila Erbereli, Jaqueline Volpe, Nicole Maia, Hélida Monteiro de Andrade, Liline Martins, Semiramis Jamil Hadad do Monte, Rafael Brandão, Adalberto Silva, Lauro Tatsuo Kubota, Dênio Souto

**Affiliations:** a Laboratório de Eletroquímica, Eletroanálise e Desenvolvimento de Sensores, Institute of Chemistry, State University of Campinas (UNICAMP), Campinas, SP 13083-970, Brazil; b Laboratório de Espectrometria, Sensores e Biossensores - Department of Chemistry, 28122 Federal University of Paraná (UFPR), Curitiba, PR 81530-900, Brazil; c Laboratório de Leishmanioses, Institute of Biological Sciences, Federal University of Minas Gerais (UFMG), Belo Horizonte, MG 31270-901, Brazil; d Laboratório de Imunogenética e Biologia Molecular, 67823 Federal University of Piaui, Campus Ministro Petrônnio Portella, Teresina, PI 64049-550, Brazil; e Faculdade de Ciências Médicas, State University of Piauí, Teresina, PI 64001-280, Brazil

**Keywords:** cryptococcosis, fungal infection, surface plasmon resonance, immunosensor, chimeric protein

## Abstract

Cryptococcosis is a severe fungal infection, particularly in immunosuppressed individuals, causing over 112,000 HIV-related deaths annually. Early and accurate diagnosis is critical, but current methods often lack the necessary sensitivity, specificity, and accessibility for point-of-care use. A major challenge is identifying highly specific bioreceptors for detecting *Cryptococcus*-specific antibodies. This study addresses these diagnostic limitations by developing a novel biosensing approach. While biosensor technology holds significant promise for rapid, sensitive, and selective responses in healthcare, effective solutions for cryptococcosis, particularly antibody detection, remain challenging. The surface plasmon resonance (SPR) technique was employed as the transduction system for constructing the biosensor. A new synthetic multiepitope protein, called protein D, was evaluated as a bioreceptor for developing an SPR immunosensor. Protein D is a chimeric protein composed of five different peptides (H18, H21, H26, S4, and Hy49) linked in specific combinations. The proposed SPR immunosensor presented limits of detection (LOD) of 0.1 μg mL^–1^ and quantification (LOQ) of 0.5 μg mL^–1^. Analysis of human sera was performed with high selectivity and reproducibility, effectively discriminating between individuals with and without cryptococcosis. To date, no plasmonic immunosensing system has been reported for detecting fungal *Cryptococcus* antibodies in human serum. In brief, this study successfully demonstrated the viability of a synthetic multiepitope protein in an SPR immunosensor for the serological diagnosis of cryptococcosis.

## Graphical abstract

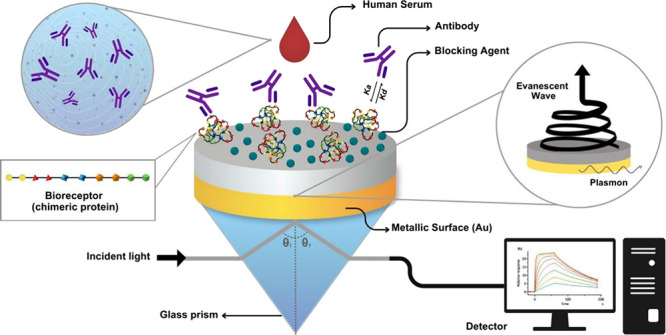

Cryptococcosis is a fungal infection commonly associated with immunosuppressive individuals, significantly affecting patients with HIV (human immunodeficiency virus). *Cryptococcus neoformans* and *Cryptococcus gattii* are the main etiological agents of this disease, which can cause meningitis and/or pulmonary diseases.^1,2^ *C. neoformans* is the leading cause of meningitis among adults living in low-income and middle-income countries, especially in sub-Saharan Africa, where HIV and AIDS (acquired immune deficiency syndrome) are the predominant risk factors. However, increasing cases in non-HIV immunocompromised and immunocompetent individuals have been reported in high-income countries.^3–7^

In 2022, the World Health Organization (WHO) listed *Cryptococcus neoformans* as a top fungal priority pathogen.^8^ Rajasingham et al. (2022) estimated that approximately 152,000 cases of cryptococcal meningitis occur annually among people living with HIV, resulting in an estimated 112,000 deaths worldwide, with 19% of deaths in AIDS patients being attributed to this fungal infection. In Brazil, studies with neurological HIV/AIDS patients have shown that cryptococcosis is the second leading cause of death, with a mortality rate of 45–65%. This high mortality underscores the urgent need for early and accurate diagnostic tools.^3,9^

Currently, the diagnosis of cryptococcosis faces significant limitations, particularly in resource-limited settings, where timely and accurate identification is critical for patient outcomes. The initial evaluation of the disease depends on clinical tests, especially in cerebrospinal fluid (CSF) and blood, mainly to diagnose meningitis caused by the *Cryptococcus* fungus, varying with microscopic, serological, and culture techniques.^10^ The analysis of the CSF commonly shows a low white blood cell count, low glucose, and elevated protein count, which may be expected in approximately 25–30% of cases and is not an accurate indicator for correctly diagnosing the disease.^2,11^ Thus, considering culture and staining, testing can present false-negative results even if the disease is widespread in the body due to its low sensitivity.

Confirmatory tests including latex agglutination, enzyme-linked immunosorbent assay (ELISA), and lateral flow assay are frequently employed.^11–13^ While ELISA and latex agglutination methods present sufficient sensitivity, their reliance on a central reference laboratory with specialized technical skills can impede timely and efficient diagnoses. Currently, the lateral flow assay stands out as the most prevalent antigen test for diagnosing cryptococcosis, primarily owing to its application in point-of-care settings. However, these platforms may be limited to application in more advanced stages of the disease as they fail to generate reliable results when there are low levels of the specific target (antigen or antibody). Notably, not all commercial lateral flow assays are capable of detecting *C. gattii* disease.^7,14^ These diagnostic shortcomings highlight a critical unmet clinical need for sensitive, rapid, and accessible methods for cryptococcosis detection, especially for early-stage disease and in diverse clinical settings.

Due to the notorious limitations regarding clinical analyses for the diagnosis of cryptococcosis, biosensor devices have emerged as promising solutions. Biosensors are devices that integrate a biological recognition element that interacts with an analyte of interest, generating a measurable signal through a transduction element.^15,16^ Notably, their sensitivity enables the detection of small concentrations of biomarkers, which may facilitate an early disease diagnosis. Moreover, biosensors exhibit remarkable agility in providing rapid responses, surpassing the turnaround times of traditional techniques. Beyond these aspects, their potential for miniaturization allows for portable, point-of-care devices, and their capability to operate with minimal sample volumes represents a significant advantage in resource-limited settings.^17^ This highlights biosensors as important tools in improving healthcare outcomes through timely and precise disease detection.^18^

While biosensors offer significant promise, their application in the diagnosis of fungal infections, particularly cryptococcosis, is an evolving field. Recent advancements have seen the development of various biosensing platforms for pathogen detection leveraging diverse transduction mechanisms. For instance, electrochemical biosensors have shown potential for rapid detection of fungal biomarkers, as demonstrated in recent studies for various fungal species.^19^ Optical biosensors, including those based on localized surface plasmon resonance or fluorescence, are also gaining traction due to their high sensitivity and real-time capabilities.^20^ Despite these developments, the specific application of biosensors for *Cryptococcus* detection, especially for antibody-based diagnostics in human serum, remains relatively underexplored compared to antigen detection or general pathogen identification. Challenges often lie in achieving the necessary specificity and sensitivity in complex clinical samples as well as the robust immobilization of biorecognition elements. This highlights the persistent need for novel and highly effective biosensing strategies tailored to the unique characteristics of cryptococcal infection.

Surface plasmon resonance (SPR) stands out among the main transducer elements due to its ability to detect and determine the specificity, affinity, and kinetic parameters from biomolecular interactions. SPR-based biosensors can be advantageous than other transducers due to their exceptional sensitivity to detect small changes in mass density on the sensor surface and the possibility of carrying out real-time analysis without the need for labeling, simplifying the experimental process, and avoiding potential interferences caused by labeling reagents. Additionally, its user-friendly operation, minimal sample preparation requirements, and straightforward, cost-effective instrumentation further highlight its potential as a valuable diagnostic tool.^18,21,22^

Given the limitations of current diagnostic methods and the high mortality associated with cryptococcosis, there is a clear rationale for exploring novel approaches for early and accurate detection. SPR-based biosensors offer a promising avenue due to their inherent advantages, including high sensitivity, real-time analysis, label-free detection, and user-friendly operation.^19,23^

In this work, a sensitive, fast, and label-free SPR immunosensor using a novel synthetic protein (named protein D), which is a chimeric protein formed by five types of peptides in different combinations, for the detection of specific *Cryptococcus* antibodies in human serum was developed. To the best of our knowledge, this is the first report on the application of an SPR-based immunosensor in cryptococcosis and the first time that this chimeric protein (D protein) has been used as a recognition element in biosensing.

## Results and Discussion

### Optimization of the Steps Involved in the Construction of the SPR Immunosensor

An optimized sensing surface was constructed by assessing the parameters of concentration and time of the bioreceptor anchoring on the previously activated 11-MUA-SAM through SPR. Given the isoelectric point (pI) of protein D close to 4, the immobilization efficacy in two different pH levels (4.0 and 7.4) was evaluated. This aimed to visualize if neutralizing the protein’s total liquid charge would influence its immobilization efficiency on the 11-MUA-SAM. From SPR analysis (Δθ_SPR_ vs. time), it was observed that the immobilization of protein D carried in phosphate buffer (PB) pH 7.4 was satisfactory. For comparison, the responses obtained at pH 7.4 (Δθ_SPR_ = 675 mdeg) were about 3 times higher than those obtained at pH 4.0 (Δθ_SPR_ = 224 mdeg). Therefore, all subsequent evaluations were conducted at a pH of 7.4. The optimal concentration of protein D was determined by assessing the sensorgram provided in Figure 1.

**Figure 1:**
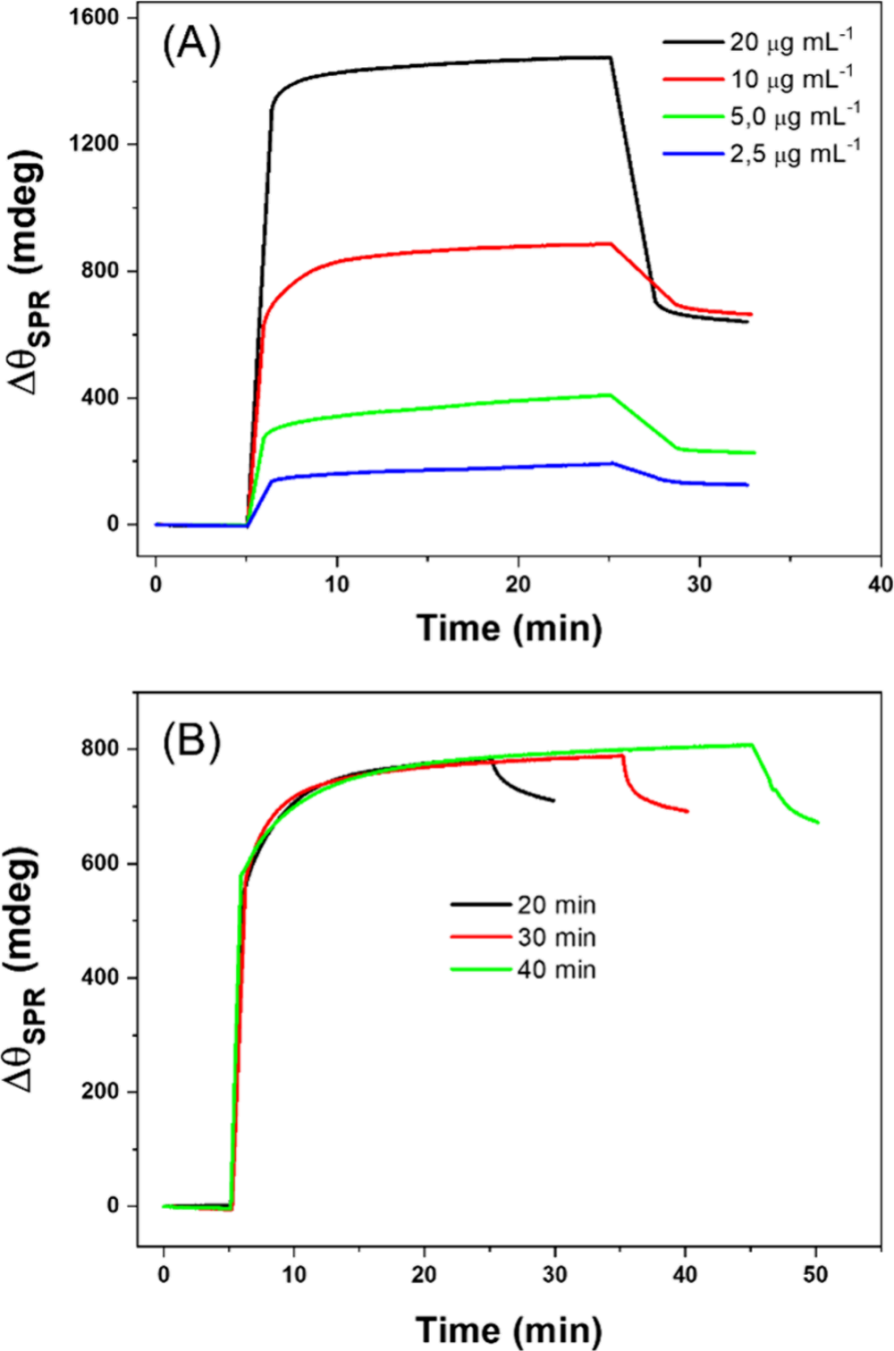
Sensorgrams obtained on a previously activated 11-MUA SAM-modified gold surface (SPR sensor chip) for real-time monitoring of protein D immobilization in PB (pH 7.4). (A) Varying protein D concentrations (20, 10, 5.0, and 2.5 μg mL^–1^). (B) Different immobilization times (10, 30, and 40 min) using a fixed protein D concentration of 10 μg mL^–1^.

In general, this sensorgram can be delineated into three distinct regions. Initially, the baseline was established by adding the PB buffer (pH 7.4) and waiting until stabilization. This demonstrates that the buffer does not interact with the functionalized surface, as evidenced by the absence of a significant change in the refractive index, indicated by a stable Δθ_SPR_. An increased SPR signal is observed in the second region upon the addition of protein D solutions at varying concentrations (from 2.5 to 20 μg mL^–1^). By adding protein D solutions to the sensor surface, it is possible to observe an intense bulk increase, which, after reaching a pseudoequilibrium behavior, constantly increases θ_SPR_ over time. By the end, upon reintroducing PB, weakly attached molecules are carried away with the buffer, leaving only the ones that strongly bind to the surface, which cause a decrease in the local refractive index, as observed by a reduction in Δθ_SPR_. This phenomenon presented a more pronounced ΔθSPR at higher concentrations, typically associated with the saturation of binding sites, which hinders the binding of new structures to the monolayer.

To define the best concentration, the covering factor (molec mm^–2^) was calculated for each concentration of protein D (Table 1). The values were obtained assuming that 1 ng mm^–2^ corresponds to Δθ_SPR_ of 120 mdeg for the total sensor area (*A*_total_ = 8.38 mm^2^).^24^ Higher concentrations, as observed for 20 μg mL^–1^, indicate response saturation, probably due to a limited number of SAM sites available for protein binding. Thus, 10 μg mL^–1^ of protein D was chosen as the concentration to construct the immunosensor.

**Table 1:** Covering Factor and Percent Surface Coverage for Each Protein Concentration Immobilized on the Sensor

**concentration** **(μg mL** ^**–1**^ **)**	**20**	**10**	**5.0**	**2.5**
covering factor (molec mm^–2^)	7.06 × 10^11^	7.33 × 10^11^	2.53 × 10^11^	1.45 × 10^11^

These findings were confirmed by utilizing the different concentrations tested and evaluating their response-ability against a positive patient’s pool (*n* = 30) diluted 400×, which contains anti-*Cryptococcus*. In this case, the use of higher concentrations did not necessarily lead to an improvement in the sensor response, likely due to saturation of the binding sites at lower concentrations.

The next step in the optimization process was the investigation of the time effect of immobilization of the chimeric protein on activated 11-MUA-SAM (Figure 1B). It is possible to observe that short immobilization times were sufficient to achieve high surface coverage, as suggested by the intensity of the SPR response as a function of time. After 20 min, a pseudoequilibrium situation may prevail, where the protein rearrangement on the functionalized metallic substrate may remain stable. In this sense, 30 min was chosen for anchoring protein D in the sensor construction stage.

### Detection of *Cryptococcus* Antibodies

Before the constructed platforms were applied to detect positive and negative human serum pools, a preliminary evaluation of the deactivation step for residual active sites was performed. Aqueous solutions of glycine (GLY) and ethanolamine (EA) were tested as quenching agents. Both effectively reduced nonspecific interactions by deactivation of residual active sites, as evidenced by significantly lower signals in negative serum samples compared with assays without this additional step. However, EA provided superior performance in preserving the signal response of positive serum samples, suggesting a lower impact on the specific interaction between the bioreceptor and antibodies in cryptococcosis patients. Consequently, EA was selected as the quenching agent for the immunosensor designed for cryptococcosis diagnosis.

The optimized platform was then further evaluated by exposing the sensor to standardized anti-*Cryptococcus* solutions with concentrations varying from 1 to 40 μg mL^–1^ prepared in PB (0.01 mol L^–1^) at pH 7.4 for 30 min. Figure 2A shows the SPR responses obtained in real time after adding different concentrations of the antibody, and the respective analytical curve is presented in Figure 2B. From these results, the following analytical parameters were obtained: limit of detection (LOD) of 0.1 μg mL^–1^ (0.9 nmol L^–1^), limit of quantification (LOQ) of 0.5 μg mL^–1^ (3.0 nmol L^–1^), and a linear range of 0.5 to 20 μg mL^–1^ with a correlation coefficient (*R* ^2^) of 0.999. For antibody concentrations above 20 μg mL^–1^, it appears that the response reaches a saturation point, likely because most receptors are unavailable for binding.

**Figure 2:**
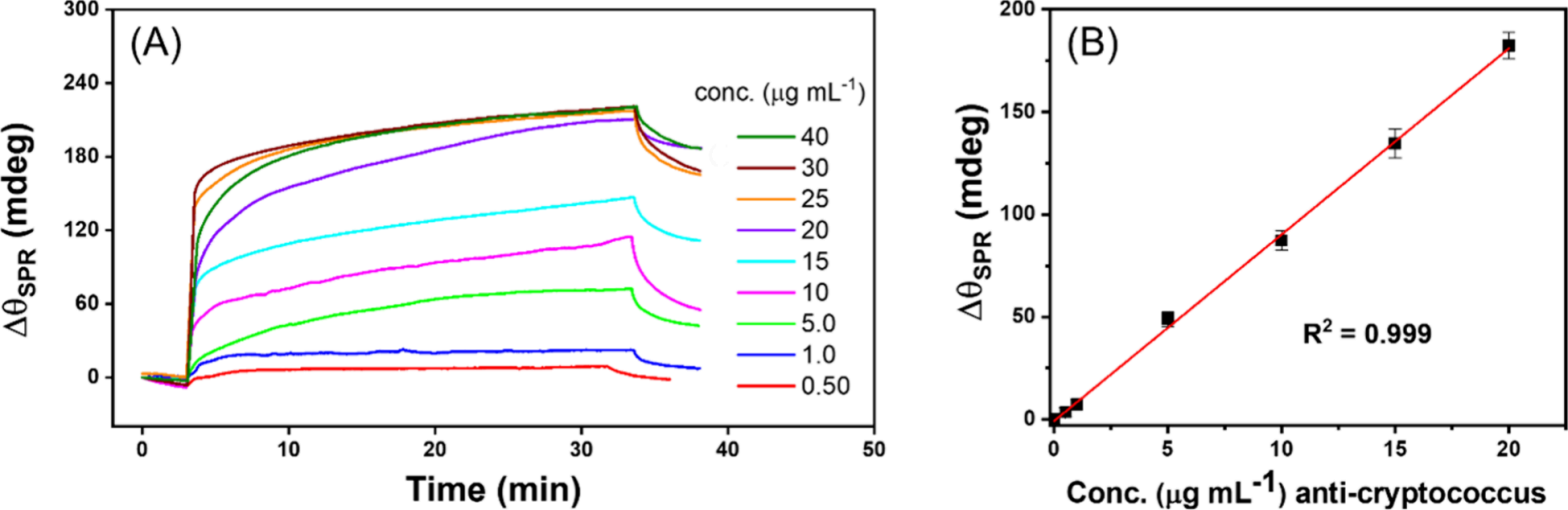
(A) Sensorgrams illustrating the association and dissociation phases for the interaction of the proposed immunosensor (protein D at 10 μg mL^–1^) with *Cryptococcus* antibodies at different concentrations (1 to 40 μg mL^–1^) prepared in PB 0.01 mol L^–1^ at pH 7.4. (B) Analytical curve obtained from the effective Δθ_SPR_ as a function of the standard specific antibody concentration (0.0, 0.5, 1.0, 5.0, 10, 15, and 20 μg mL^–1^) with a correlation coefficient (*R* ^2^) equal to 0.999.

Since protein D is a multiepitope bioreceptor derived from six immunogenic and hypothetical proteins, it may interact with various polyclonal antibodies present in patient serum. Therefore, establishing a reliable calibration curve for quantitative antibody analysis would require additional studies and methodological optimizations due to its complexity. As a result, the proposed approach should be considered semiquantitative. While the signal correlates with antibody concentration in the sample matrix, it lacks the precision necessary for a fully quantitative assessment.

### Application of the Immunosensor toward Human Serum Samples from the Positive and Negative Groups for Cryptococcosis

For these analyses, a pool obtained from positive human serum (*n* = 30) and another one from negative human serum (*n* = 30) for cryptococcosis were diluted in different dilution factors (1:50 to 1:6400) and detected in real time by the proposed SPR-based immunosensor. Figure 3 shows the responses obtained for the positive (Figure 3A) and negative (Figure 3B) pool samples.

**Figure 3:**
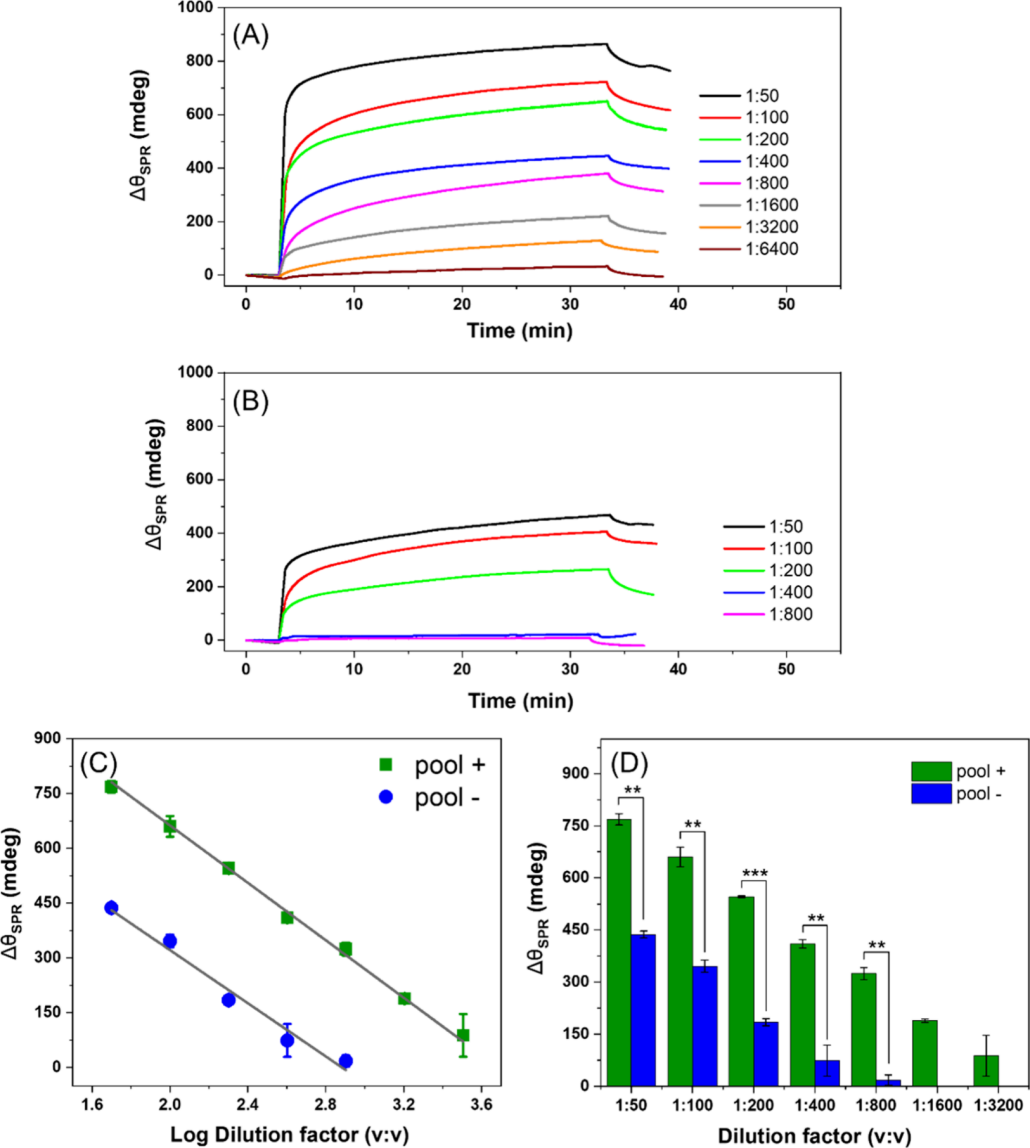
Sensorgrams illustrate the association and dissociation phases obtained by the SPR immunosensor upon the addition of positive (A) and negative (B) human serum samples for cryptococcosis. For these analyses, positive (*n* = 30) and negative (*n* = 30) serum pools were diluted in PB (pH 7.4) at various volume-to-volume ratios (1:50, 1:100, 1:200, 1:400, 1:800, 1:1600, 1:3200, and 1:6400). The response intensities obtained after injection of the positive and negative serum pools are shown in panel C as a linear correlation (*R* ^2^ > 0.98) between the effective Δθ_SPR_ and the logarithm of the dilution factor. (D) Effective Δθ_SPR_ values as a bar graph against the dilution factor (*n* = 2 replicates).

It is possible to observe a significantly higher response for the positive pool than for the negative one, demonstrating the immunosensor’s capability as a novel methodology for cryptococcosis diagnosis. At lower serum dilutions, a high Δθ_SPR_ is observed due to the complexity of the serum matrix, which contains numerous components capable of nonspecifically binding to the functionalized gold surface, leading to a fouling effect. To minimize this interference, serial dilutions are performed until the response from the negative serum pool becomes negligible. This strategy enables the interpretation of the positive signal as primarily due to the presence of specific antibodies interacting with the bioreceptor.

Comparing the responses obtained, a wide detection range for the positive sample was observed. Conversely, from a dilution of 1:800 onward, no significant response for the negative pool was detected, demonstrating the excellent selectivity of the immunosensor. This is probably due to the ability of the chimera protein (protein D) to react preferentially with cryptococcal antibodies.

For all tested dilutions, the positive responses differed significantly from the negative controls (*p* < 0.01) (Figure 3D). This indicates that the bioreceptor was able to specifically interact with anti-*Cryptococcus* present in the serum samples. Moreover, this interaction was consistently reproducible across the dilution range, exhibiting a strong linear correlation between the effective Δθ_SPR_ and the logarithm of the dilution factor for the positive samples (*R* ^2^ > 0.998) as well as for the negative samples (*R* ^2^ > 0.987). The logarithmic relationship is expected due to the saturation of recognition sites at higher concentrations. Additionally, the increase in the standard deviation observed in more diluted samples is likely related to the propagation of uncertainty inherent in serial dilutions.

At a 1:800 dilution, the chimeric protein produced an almost negligible response against the negative sera (18 ± 15 mdeg) (Figure 3D). In contrast, the positive sera at the same dilution elicited a Δθ_SPR_ response nearly 20-fold higher (324 ± 17 mdeg), which is expected to arise predominantly from specific interactions, given the minimal response observed in the negative pool at this dilution. In the analytical curve shown in Figure 2B, a saturated response was observed at approximately 180 mdeg. This discrepancy can be attributed to the nature of the bioreceptor, which can interact with multiple antibody types, such as IgG and IgM. These antibodies differ in molecular mass and can influence the refractive index in distinct ways, unlike the monoclonal antibody used in the analytical curve.

In general, one of the most widely used portable diagnostic approaches for cryptococcosis is the LFA, with a well-established commercial test available for point-of-care (POC) diagnosis based on semiquantitative or qualitative antigen detection. LFAs can be applied to CSF, serum, or urine samples, typically showing improved sensitivity and selectivity when used with CSF. However, CSF collection is invasive and therefore more difficult to implement in clinical trial settings.^25^ Considering the diagnosis of cryptococcosis, a cost-effective platform is essential for technology transfer to real-world applications.

In this context, classical SPR analysis may present some limitations when compared to other techniques, such as immunochromatographic-based tests, which are low-cost, easy to use, and portable.^26^ However, SPR remains one of the most effective techniques for exploring and studying new biomolecular interactions due to its high sensitivity, label-free detection, and real-time analysis.^27^ These characteristics position SPR as a key approach for characterizing novel bioreceptors, thereby facilitating the development of high-performance and reliable diagnostic tools. Moreover, the integration of smartphone-based SPR platforms with AI-enhanced signal processing holds great promise for the development of diagnostic biosensing devices tailored to POC applications, possibly offering quantitative results, a potential advantage over traditional LFAs.^28,29^

Regardless of the transducing technique employed, this study also explored the use of a novel bioreceptor for the identification of antibodies in serum, an unconventional approach in the context of cryptococcosis diagnosis. This is mainly due to the typically suppressed immune response observed in advanced HIV patients, as well as the lack of specific antigens capable of providing selective responses.^30^ Very few studies in recent years have investigated specific antibodies as targets for cryptococcosis diagnosis using biosensors. Instead, DNA detection, representing a molecular approach, has been more commonly explored than serological alternatives (Table 2).

**Table 2:** Examples of Cryptococcosis Biosensors Recently Reported^a^

**method**	**platform**	**receptor**	**target**	**sample**	**LOD**	**ref**
enzyme-linked amperometric amplification	BSA-Au	dsDNA	DNA	cerebrospinal fluid	800 fmol L^–1^ (*C. neoformans*)	Liu et al., 2018 ^31^
SERS	AgNPs			cerebrospinal fluid extracted colony		Hu et al., 2020 ^32^
LFA		CRISPR-Cas12a	DNA	cerebrospinal fluid	10^2^ copies μL^–1^ (*C. neoformans* and *C. gattii*)	Liu et al., 2024 ^26^
electric microfluidic (DPV)	rGO/AuNPs	DNA probe	DNA	extracted DNA	60 pg mL^–1^ (*C. neoformans*), 100 pg mL^–1^ (*C. gattii*)	Kong et al., 2024 ^33^
fluorescence	DEMA	CRISPR-Cas12a	DNA	extracted DNA	0.5 pmol L^–1^ (*C. neoformans* and *C. gattii*)	Tong et al., 2024 ^34^
SPR	MUA-Au	protein D	antibodies	serum	0.1 μg mL^–1^ (*C. gattii*)	this work

a Abbreviations: AgNPs, silver nanoparticles; Au, gold; AuNP, gold nanoparticles; BSA, bovine serum albumin; DEMA, deep learning-enhanced microwell array; DPV, differential pulse voltammetry; LFA, lateral flow assay; MUA, mercaptoundecanoic acid; rGO, reduced graphene oxide; SERS, surface-enhanced Raman scattering; and SPR, surface plasmon resonance.

By employing a multiepitope protein (protein D), this study may pave the way for improved quantitative analysis through SPR-based strategies, potentially enhancing epidemiological monitoring and enabling early-stage seroconversion detection through antibody detection. Also, the plasmonic immunosensor based on synthetic multiepitope proteins holds promise for preventive therapies in humans and could enable the detection of asymptomatic carriers in animals, representing a valuable tool for the control of cryptococcosis.^35^ However, further studies are needed to evaluate the applicability of this device in low-resource settings, its selectivity against other fungal infections, and its sensitivity for antibody detection in immunosuppressed and immunocompetent individuals.

## Conclusions

This study may reveal new possibilities for the development of artificial bioreceptors, such as multiepitope protein D, for application in biosensors targeting the diagnosis of fungal diseases, an area that still lacks reliable, affordable, and portable diagnostic tools. The potential of this bioreceptor could also be explored by using alternative transduction strategies, such as lateral flow assays (LFAs) and electrochemical methods. Furthermore, combining miniaturized surface plasmon resonance (SPR) platforms with machine-learning-based data analysis could support the development of portable point-of-care devices. This approach would take advantage of the high sensitivity of SPR, known as the gold standard for investigating biomolecular interactions, while enhancing its applicability in user-friendly, real-world diagnostic solutions

The most critical steps required for sensor construction were optimized, such as pH (7.4), concentration (10 μg mL^–1^), and immobilization time (30 min) of the chimera protein on a SAM of 11-MUA previously formed on an SPR sensor chip (gold surface). In the quenching step of nonspecific sites on the previously biofunctionalized surface, the best result was obtained with an ethanolamine aqueous solution (100 mM), which significantly reduced the interaction of nonspecific biomolecules with the sensor.

From the responses obtained by the proposed SPR immunosensor for the detection of purified anticryptococcal solutions, the following analytical parameters were obtained: limit of detection (LOD) of 0.1 μg mL^–1^ (0.9 nmol L^–1^), limit of quantification (LOQ) of 0.5 μg mL^–1^ (3 nmol L^–1^), and a linear range of 0.5 to 20 μg mL^–1^ with a correlation coefficient (*R* ^2^) of 0.999. Afterward, the application of the SPR immunosensor for the detection of *Cryptococcus* antibodies in human serum samples evidenced the high selectivity and reproducibility of the proposed method, perfectly discriminating individuals from positive and negative groups for cryptococcosis. In addition to the intrinsic technology involved in the transduction principle of the SPR equipment used, these results are also due to the ability of this new chimeric protein (synthetic protein) to react preferentially with the *Cryptococcus* antibodies.

Therefore, this study highlights the feasibility of SPR technology, emphasizing the development of an immunosensor exploiting a synthetic protein as a recognition element for the simple, rapid, and reliable serological diagnosis of cryptococcosis. This study may reveal new possibilities for the application of SPR biosensors in the diagnosis of fungal diseases, including the development of portable devices for point-of-care applications.

## Materials and Methods

### Chemicals

*N*-(3-Dimethylamino-propyl)-*N*-ethylcarbodiimidehydro-chloride (EDC), *N*-hydroxysuccinimide (NHS), 11-mercaptoundecanoic acid (11-MUA), ethanolamine (EA), and glycine (GLY) were purchased from Sigma-Aldrich Chemical (St. Louis, MO, USA). Ethylenediaminetetraacetic acid (EDTA), KCl, KOH, ethanol (99%), and monobasic sodium phosphate were obtained from LabSynth LTDA (SP, Brazil). The 11-MUA ethanolic solution and aqueous solutions of EA, GLY, and the EDC/NHS mixture were prepared before use. The phosphate buffer (PB, 0.01 mol L^–1^, pH 7.4), used in this work, was prepared by mixing equimolar amounts of KH_2_PO_4_ and Na_2_HPO_4_ (0.005 mol L^–1^ each), with 0.1 mol L^–1^ KCl added, and the pH adjusted using NaOH. Deionized water purified and obtained from a Milli-Q system (Millipore) was used to prepare the solutions.

### Apparatus

To evaluate in real time the immobilization of the chimeric protein (antigen) and the antigen–antibody interactions, measurements were performed by an SPR Autolab Spirit instrument (Eco Chemie B.V., Utrecht, The Netherlands) under static conditions. The optical system consisted of a glass prism and a planar gold SPR disk, which were both obtained from Xantec Bioanalytics (Muenster, Germany). The equipment has a laser diode with a wavelength fixed at 670 nm. This system is equipped with a cuvette, and its operation mode is based on the Kretschmann configuration. All experiments were conducted at 23 ± 1 °C in PB (0.0.1 mol L^–1^) at pH 7.4.

### Biological Samples

The synthetic antigenic protein (protein D) is a chimeric multiepitope molecule designed using validated immunoreactive peptides derived from *C. gattii* proteins, identified through immunoinformatics and immunoproteomics tools, which suggested novel antigen candidates for the diagnosis of cryptococcosis.^35–37^ Protein D showed satisfactory reactivity against the serum of cryptococcosis patients and was derived from six immunogenic proteins for *C. gatti*, Hsp70, GrpE, sks2, enolase, and two conserved hypothetical proteins, CGNB 1302 and CGNB 1079.^37^ This recombinant multiepitope protein comprises five different peptides (H18, H21, H26, S4, and Hy49) linked in a specific sequence,^35^ as described in Table 3, along with its predicted molar weight (MW) and isoelectric point (pI).^37^

**Table 3:** Sequence of Peptides (H18, H21, H26, S4, and Hy49) of Protein D, with Its Respective Predicted Molar Weight (MW) and Isoelectric Point (pI)

protein D peptide sequence	**pI**	MW (10^3^ g mol^–1^ **= kDa)**
(−H18–H18–H21–H21–H26–H26–S4–S4–Hy49–Hy49−)_*n*_	4.05	35.4

ELISA (enzyme-linked immunosorbent assay) was performed using protein D to determine the concentration of the specific IgGs to protein D, obtaining a purified concentration of *Cryptococcus*-specific antibodies (0.5 to 50 μg mL^–1^) dissolved in PB solution at pH 7.4

Human serum samples with positive (*n* = 30) and negative (control group, *n* = 30) confirmed diagnosis for cryptococcosis were used in different dilutions in PB solution at pH 7.4. The experiments with these samples were carried out following the guidelines of the Human Ethics Committee at the State University of Piau (protocol number 079/2008).

### SPR-Based Immunosensor: Construction and Application

#### Construction

The SPR-based immunosensor was constructed by using the following steps, according to the scheme in Figure 4.

**Figure 4:**
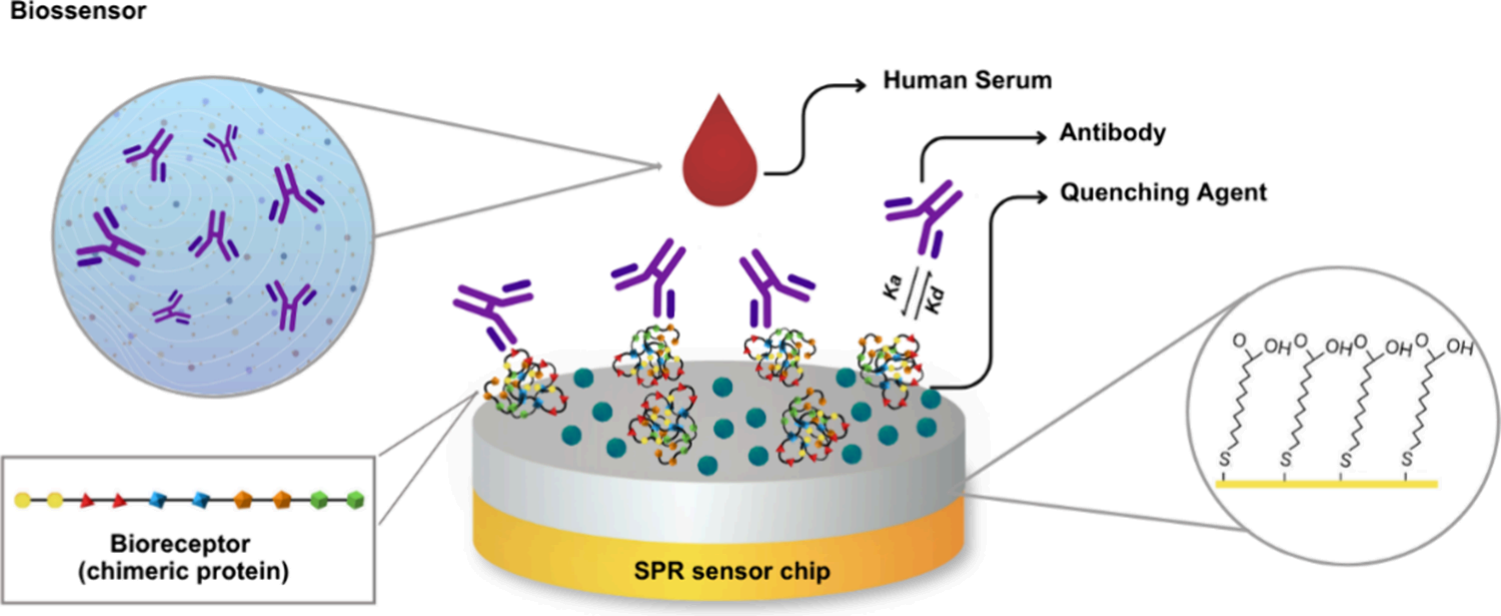
Schematic representation of the SPR immunosensor: a gold disc (SPR sensor chip) coated with 11-MUA SAM, which is activated via EDC/NHS for covalent antigenic protein immobilization; nonspecific reaction site deactivation with ethanolamine; and detection step (antigen–antibody interaction).

Initially, the gold surface of the SPR sensor chip was cleaned by immersion in piranha solution (H_2_O_2_/H_2_SO_4_ in a 1:3 ratio) for 3 min followed by sonication in acetone and isopropanol for 5 min each. Between each step, successive washes with deionized water were performed, and by the end, the sensor chips were dried under a nitrogen flow. Following the cleaning step, the SPR disk was functionalized by forming a self-assembled monolayer (SAM) through submersion of the SPR sensor chip in an ethanolic 11-MUA (1.0 mmol L^–1^) solution overnight at 20 °C. For the immobilization of protein D, the terminal carboxylic groups of 11-MUA were activated via an aqueous solution of EDC (150 mmol L^–1^)/NHS (100 mmol L^–1^) for 10 min. The immobilization parameters of this chimeric protein were optimized through SPR measurements, considering the concentration, reaction time, and pH. After the immobilization step, an additional procedure was carried out to deactivate remaining reactive sites by evaluating two different quenching agents and their influence on the biosensor response to both positive and negative pooled human serum. In this step, glycine (GLY) and ethanolamine (EA) were applied ex situ for 5 min. Control measurements were also performed in the absence of any quenching agent. Both EA and GLY were prepared in an aqueous solution at a concentration of 100 mmol L^–1^.

#### Application

After optimizing the conditions involved in the step of construction of the immunosensor, the next step consisted of adding purified solutions with known concentration of antibodies (anti-*Cryptococcus*) to evaluate the specific interaction between the chimeric protein D and specific immunoglobulin G of the cryptococcosis. In this step, the limits of detection (LOD) and quantification (LOQ) were calculated by using eqs 1 and 2, respectively.

$$\mathrm{LOD}=3.3\times \frac{\sigma }{S}$$ 1

$$\mathrm{LOQ}=10\times \frac{\sigma }{S}$$ 2

in which σ is the standard deviation of the response for blank measurements and *S* is the slope of the calibration curve.

Afterward, the proposed immunosensor was applied to assess the ability of the studied chimeric protein (protein D) to identify antibodies against *Cryptococcus* in human serum samples. For this, a positive pool was prepared by utilizing 30 individual serums randomly selected from the cryptococcosis patient samples against a control group (negative pool), which contained 30 sera of healthy patients. These samples were serially diluted in 0.01 mol L^–1^ PB (pH 7.4), with dilution ratios ranging from 1:50 to 1:3200 (v/v).

All statistical analyses were performed using the OriginPro software (OriginLab, MA, USA) and evaluated by *t* tests with a 95% confidence interval. Statistical differences were indicated by * for *p* < 0.05, ** for *p* < 0.01, and *** for *p* < 0.001.

## †

In memoriam.

## Abbreviations

11-MUA-SAM: 11-mercaptoundecanoic acid self-assembled monolayer
CSF: cerebrospinal fluid
EA: ethanolamine
EDC: 1-ethyl-3-(3-(dimethylamino)­propyl)­carbodiimide
ELISA: enzyme-linked immunosorbent assay
Gly: glycine
LOD: limit of detection
LOQ: limit of quantification
MUA: 11-mercaptoundecanoic acid
NHS: *N*-hydroxysuccinimide
SAM: self-assembled monolayer
SPR: surface plasmon resonance
UNAIDS: Joint United Nations Programme on HIV/AIDS
